# Genetic Diversity in the Orenburg Goat Breed Revealed by Single-Nucleotide Polymorphism (SNP) Analysis: Initial Steps in Saving a Threatened Population

**DOI:** 10.3390/genes15111375

**Published:** 2024-10-25

**Authors:** Tatiana E. Deniskova, Arsen V. Dotsev, Alexandra S. Abdelmanova, Sergey N. Petrov, Alexey N. Frolov, Stanislav A. Platonov, Elena A. Gladyr, Igor V. Gusev, Marina I. Selionova, Andrey N. Rodionov, Svyatoslav V. Lebedev, Darren K. Griffin, Michael N. Romanov, Natalia A. Zinovieva

**Affiliations:** 1 L. K. Ernst Federal Research Center for Animal Husbandry, Dubrovitsy, Podolsk 142132, Moscow Oblast, Russia; horarka@yandex.ru (T.E.D.); asnd@mail.ru (A.V.D.); preevetic@mail.ru (A.S.A.); citelekle@gmail.com (S.N.P.); elenagladyr@mail.ru (E.A.G.); igorgusev@mail.ru (I.V.G.); rodiand@yandex.ru (A.N.R.); n_zinovieva@mail.ru (N.A.Z.); 2 Federal Research Center for Biological Systems and Agrotechnologies, Russian Academy of Sciences, Orenburg 460000, Orenburg Oblast, Russia; forleh@mail.ru (A.N.F.); platonstas1994@mail.ru (S.A.P.); lsv74@list.ru (S.V.L.); 3 Subdepartment of Animal Breeding, Genetics and Biotechnology, Russian State Agrarian University—Moscow Timiryazev Agricultural Academy, Timiryazevskaya Street, 41, Moscow 127434, Russia; selionova@rgau-msha.ru; 4 School of Biosciences, University of Kent, Canterbury CT2 7NJ, UK; d.k.griffin@kent.ac.uk; 5 Animal Genomics and Bioresource Research Unit (AGB Research Unit), Faculty of Science, Kasetsart University, Chatuchak, Bangkok 10900, Thailand

**Keywords:** Orenburg goats, single-nucleotide polymorphism (SNP), local breeds, population structure, genetic diversity

## Abstract

**Background/Objectives**: Orenburg goats are renowned for their soft down that acts as a substrate for warm clothing, particularly shawls that have an international reputation. As with many local livestock breeds, however, the Orenburg is presently at risk of extinction, an issue that can be addressed by assessing population genetic diversity and, thereafter, encouraging as much outbreeding as possible. Using single-nucleotide polymorphism (SNP)-based data, therefore, we analyzed the genetic diversity and population structure of modern Orenburg goats using samples collected from an expedition to Orenburg Oblast in 2024. **Methods**: We applied the Goat SNP50 BeadChip (Illumina, San Diego, CA, USA) for the genotyping of Orenburg goats from modern and archived populations. SNP genotypes of three Orenburg populations sampled in 2017 and 2019, Altai Mountain, Altai White, and Soviet Mohair breeds, were added to the dataset. **Results**: Principal component analysis and network and admixture analyses demonstrated that the genetic background inherent to the archived group of Orenburg goats was maintained in all modern populations. Values of genetic diversity indicators in modern populations were compatible with those obtained in comparison groups. Runs of homozygosity (ROH) were found in all the Orenburg goat populations (with a mean ROH length of 72.6–108.9 Mb and mean ROH number of 28–36). Genomic inbreeding based on ROH was low in all the Orenburg populations (*F*_ROH_ = 0.03–0.045). **Conclusions**: We showed that the ancestral background is retained in present-day Orenburg goats sampled in 2024. We provide the genetic basis through which certain breeder animals may be selected and bred traditionally or ex situ through a conservation program of gamete preservation.

## 1. Introduction

Down knitting, a traditional handicraft throughout Russia, has developed as a cultural phenomenon, in part because of the local continental climate in certain regions [1]. Mittens, socks, and kneepads knitted from homespun wool tend to predominate [1,2]. The value of down from the Orenburg goat breed was first described at the end of the 18th century. Unlike many types of sheep wool, it remained unique to the Orenburg region, with some historical reports referring to the fact that goats were easier to keep there than sheep [3]. In addition, clothing produced by the local communities from goat down that was deemed to be soft and of good quality was a major driver of the economy in the Orenburg region [4].

The rise of the down-knitting industry in Orenburg Province began with the establishment of the city of Orenburg as a fortress in 1743. The settlers, mainly Cossacks from Central Russia and the Volga regions, arrived at the new province previously inhabited by semi-nomadic peoples (i.e., Tatars, Bashkirs and Kazakhs) [5]. According to the historical reports, girls from as young as seven years old, plus many women, were occupied in their free time by down knitting in all Cossack villages from Orsk to Orenburg [6].

Starting by producing warm clothing elements, Orenburg handicraft has reached a higher level of artistry by implementing the openwork down knitting. This created the famous Orenburg openwork shawls called “pautinka”, “gossamer webs”, “spider web” and “wedding ring”, made by combining the Orenburg goat down with warp threads of cotton or silk. They received a special status in the world history of decorative art as unique artistic phenomena [7]. Orenburg openwork shawls, in particular, gained worldwide attention by the middle of the 19^th^ century [8].

Emily Jackson, the author of *A History of Hand-Made Lace* (1900), noted that the artistic feeling and individuality of the lace-maker and attention to detail in the labor were more valuable elements of lace-making industry than the starting materials [9]. This drew parallels with openwork down knitting in this regard as, for the down knitter, it was a hard and painstaking labor, with people often spending up to nearly 200 h making an openwork shawl [10]. Unlike in lace-making, however, Orenburg openwork down knitting owed its origin and popularity, at least in part, to the best-quality down produced by the local Orenburg goats.

Twelve traditional patterns of Orenburg openwork shawls [11] are widely known outside the Southern Urals. These were inspired by nature as reflected in their names such as “millet”, “kosoryadki” (or “inclined rows”), small and large “gluhotinki” (or “blackberry and black currant motifs”), “mouse trail”, “cat’s paws”, “bead path”, “peas”, “fish”, “accordions”, “honeycombs” and “korol’ki” (“round bead necklace”) [11]. In addition, new non-traditional elements such as “peacock”, “lilies of the valley” and “cones” were also well established [12].

The Orenburg goat breed was created by a long-term selection of native goats with fine elastic fibers in Orenburg Province [13]. Winter freezing snowstorms with frequent strong winds and scorching summer heat played an important role in the formation of this breed. In all likelihood, the development of an undercoat and light down in these goats was associated with fitness and protection from unfavorable local climate-specific conditions because the undercoat lost its lightness and ability to retain heat in other locations [10]. Goat down is called “soft gold” because no other type of yarn is quite so soft, light and warm [10]. In addition, the down produced by Orenburg goats can become fluffy, giving products made from it a particular softness and perceived beauty [13].

The first goat breeding state farm in the Orenburg region was founded in 1932 in the floodplain of the Guberlya River on the southern spurs of the Ural Mountains [13]. Purebred goats were improved by selection based on coat phenotypes. That is, animals with a dark-gray coat and finer down fibers were preferred. To obtain a higher down yield, crossings with Don males have been carried out since 1937. Crossbred goats, however, lost many of the most valuable qualities prized in Orenburg down, such as fineness, elasticity, softness, uniform color and resilience [14].

In the second half of the 20th century, the population of down goats in Russia was one of the largest in the world [13]. Despite this, the national recognition of the Orenburg breed specifically went into decline. This decline has its roots from around 1990, with the reduction in state and regional financial support for down goat breeding. Throughout the 20th century, the down knitting industry faced increasing economic unprofitability, with Orenburg goat breeding particularly hit because of the increased use of synthetic fibers and cheaper down from Central Asia (i.e., Uzbekistan and Kyrgyzstan). The latter were used for the production of imitations and counterfeits of Orenburg shawls. Moreover, increased costs of animal feeding and maintenance resulted in a decrease in the Orenburg down quality. In addition, economic globalization and changes in fashion trends caused a significant narrowing of the sales markets for traditional down-knitting products [10]. The combined influence of these factors thereby led to a dramatic decrease in the total number of Orenburg goats. Consequently, ~111,700 goats of this breed were registered in Russia in 1991 [15], falling to ~16,900 goats by 2000 [16]. According to the latest official records, the population size of the Orenburg breed decreased to ~3400 goats, including ~2800 in Orenburg Oblast (formerly Orenburg Province) and only 600 or so in the Republic of Bashkortostan [17]. In 2022, the breeding stock of the Orenburg breed was solely at the Donskoy Agricultural Production Company (collective farm). By 2023, however, not a single farm for breeding the Orenburg goats was left in Russia [17].

Recent genetic studies have shown that utilizing single-nucleotide polymorphism (SNP) genotyping for local livestock species can be very helpful in understanding their current population structure and in elucidating probable admixtures. For instance, according to Dadousis et al. [18], SNP genotyping helped define the population genetic diversity of the Garfagnina goat breed that has a distinct gene pool compared to other Italian breeds, while, up to that point, it was threatened with extinction. Furthermore, an analysis of modern, archived and museum samples of the Livni pig breed demonstrated that historical genomic backgrounds found from local Russian livestock populations have been admixed with commercial breeds in order to improve them massively [19]. Comparative investigations of local and commercial breeds may have wider implications in influencing national conservation priorities. Examples include the study by Wang et al. [20], addressing total genetic and allelic diversities in the Chinese Guangfeng and Ganxi goat breeds, and that by Adeniyi et al. [21], estimating the diversity parameters of Kosovar Balusha sheep and highlighting the urgency of preserving this local breed clearly distinguished from other sheep of Southeastern Europe (Bardhoka, Ruda and Pramenka). On the other hand, a genomic assessment of Czech and Slovak local dairy goats demonstrated that two breeds (White Shorthair and Brown Shorthair) were not clearly genetically distinguishable [22]. In general terms, estimating the genetic diversity (or lack of it in the latter case [22]) using SNP arrays allows, therefore, for the selection of breeder pairs that are the most genetically distinct from one another, thereby increasing hybrid vigor and thus the survivability of individuals and populations. Such a strategy is particularly valuable in endangered livestock breeds, i.e., those being at risk of extinction.

In 2024, the newly established National Center for Genetic Resources of Agricultural Animals (NCGRAA) at the L. K. Ernst Federal Research Center for Animal Husbandry (LKEFRCAH) launched a mission to promote the conservation of local livestock genetic resources by the cryopreservation of different types of germplasm and by breeding ex situ. To attain the ultimate goal of the conservation of the gene pool of Orenburg goats, the combined use of genetic and reproductive technologies is essential. Two fundamental objectives, therefore, need to be achieved. The first one is to collect samples of Orenburg goats representing the contemporary gene pool of this breed. This goal has already been accomplished successfully thanks to a specially organized expedition to smallholders’ farms, where live animals are raised. The second goal is the genome-wide evaluation of these specimens to select typical animals that will be used to preserve the original genetic background of the Orenburg breed. Therein lies the purpose of this study, to perform a comparative analysis of Orenburg goat populations representing the contemporary gene pool based on SNP genotyping data.

## 2. Materials and Methods

### 2.1. Ethics Statement

The animals used in the current study were treated and all relevant procedures were conducted according to the LKEFRCAH ethical guidelines following Protocol No. 2 that was approved by the LKEFRCAH Commission on the Ethics of Animal Experiments and dated 15 May 2024. The tissue samples (fragments of the auricle) of goats from modern populations of the Orenburg breed were collected by trained personnel using tagging forceps for livestock under strict veterinary rules in accordance with those for executing laboratory research (tests) in the implementation of the veterinary control (supervision) as approved by Eurasian Economic Commission Council Decision No. 80 (dated 10 November 2017) during the work on the goat herd in 2024.

### 2.2. Sample Collection and DNA Extraction

Samples consisted of populations of the Orenburg breed corresponding to different generations as listed in Table 1.

**Figure 1:**
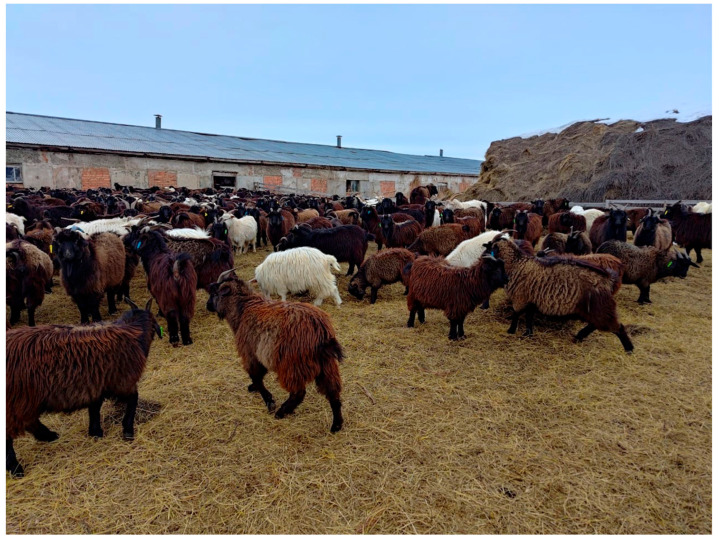
Representatives of modern goats of the Orenburg breed. A herd kept in Orenburg Oblast. (Image source: authors’ own photograph).

Accordingly, we collected tissue samples (i.e., fragments of the auricle) of the ORN_S_2024_g, ORN_F_2024_g and ORN_F_2024_w populations from two farms in Orenburg Oblast in 2024 (Figure 1) within the framework of the activities of the LKEFRCAH NCGRAA.

Blood samples from the ORN_D_2012_g archived group were obtained from the LKEFRCAH Collection of Animal Genetic Resources.

SNP genotypes of goats from the Altai Mountain (*n* = 34), Altai White (*n* = 27), Soviet Mohair (*n* = 35) and three populations of the Orenburg breed (ORN_D_2017_w, ORN_G_2017_g and ORN_D_2019_g) were generated previously [23]. We used SNP genotypes of the Altai Mountain, Altai White and Soviet Mohair breeds as a comparison group.

### 2.3. SNP Genotyping and Quality Control

Genomic DNA was isolated from tissue samples of goats from the groups ORN_S_2024_g, ORN_F_2024_g and ORN_F_2024_w using the DNA Extran 2 kit (Syntol, Moscow, Russia) and from the archived blood of goats from ORN_D_2012_g using the DNA Extran 1 kit (Syntol, Moscow, Russia).

To test the quantity and quality of the DNA, the concentration of double-stranded DNA was measured using a Qubit™ fluorometer (Invitrogen, Life Technologies, Carlsbad, CA, USA), and the absorption at 260 and 280 nm (OD 260/280) was determined using a NanoDrop 8000 instrument (Thermo Fisher Scientific Inc., Waltham, MA, USA).

Goats were genotyped using the Illumina Goat SNP50 BeadChip (llumina, San Diego, CA, USA) [24,25].

Quality control was performed by setting a cutoff of 0.5 for the GenCall and GenTrain scores [26]. Samples for which less than 90% loci (i.e., with the quality control setting --mind 0.1) were genotyped were removed from the analysis. We also excluded SNPs for which less than 90% (--geno 0.1) of the samples were called in, those with a minor allele frequency (MAF) lower than 5% (--maf 0.05), and those located on sex chromosomes and with unknown positions.

A total of 49,941 SNPs were genotyped; after data filtering (quality control), 47,625 SNPs were utilized for further analysis.

### 2.4. Genetic Relationship and Population Structure

Pairwise values of genetic differentiation (*F*_ST_) [27] were estimated using the R package StAMPP (Version 1.6.3) [28]. Pairwise identity-by-state (IBS) distances were calculated using PLINK v1.9 [29] (with the software setting --distance 1-ibs). The Neighbor-Net graphs based either on the matrix of pairwise *F*_ST_ values for all studied goat groups or IBS distances for the Orenburg populations were plotted using SplitsTree 4.14.5 software [30].

Principal component analysis (PCA) was performed using PLINK v1.9 [30] and visualized using the R package ggplot2 (Version 3.5.1) [31]. For a better understanding of genetic relations between Orenburg goats, an additional PCA was performed separately for seven Orenburg goat populations without the comparison group.

Cluster analysis was performed using Admixture v1.3 software [32] and plotted using the R package pophelper (Version 2.3.1) [33]. The choice of the optimal number of ancestral populations (K) was based on the lowest cross˗validation (CV) error compared to other K values as implemented in a standard Admixture CV procedure [32].

We also calculated *F*_ST_ values for each SNP, comparing the modern Orenburg populations and the comparison group to define specific SNPs.

### 2.5. Genetic Diversity Analysis

Genetic diversity indicators including the observed heterozygosity (*H_O_*), unbiased expected heterozygosity (*_U_H_E_*) [34], rarefied allelic richness (*A_R_*) [35] and inbreeding coefficient (*_U_F*_IS_) based on *_U_H_E_* with a 95% confidence interval (CI 95%) were calculated using the R package diveRsity (Version 1.9.90) [36].

### 2.6. Runs of Homozygosity and Genomic Inbreeding

We used a consecutive runs method [37] implemented in the R package detectRUNS [38] to estimate runs of homozygosity (ROH) parameters. An acceptable ROH permitted up to one potential heterozygous genotype and one SNP with a missing genotype; 500 kb was the minimum ROH length.

The minimum number of SNPs (*l*), first suggested by Lencz et al. [39] and later adopted by Purfield et al. [40] in a study on cow breeds, was computed in order to reduce false positive results as follows:

$$l=\frac{{log}_{e}\frac{\alpha }{n_{s}\;\cdot \;n_{i}}}{{log}_{e}(1\;-\;\bar{het})},$$

where *n_s_* means the number of genotyped SNPs per individual, *n_i_* denotes the total number of genotyped animals, *α* is the false positive ROH percentage (which we set at 0.05), and $\bar{het}$ represents the mean heterozygosity for all SNPs. The calculated *l* was equal to 17.

We estimated ROH for each goat and then categorized ROH in the following length classes: 1–2 Mb, 2–4 Mb, 4–8 Mb, 8–16 Mb and >16 Mb.

For every breed and length group, the total number of detected ROH was computed for each individual. By adding the total ROH length for every goat in the populations and averaging the outcomes per breed group, the mean sum of ROH was calculated.

The genomic inbreeding coefficient based on ROH (*F*_ROH_) was computed as the sum of the length of all ROH per goat proportioned to the total autosomal SNP coverage.

## 3. Results

### 3.1. Genetic Differentiation

**Figure 2:**
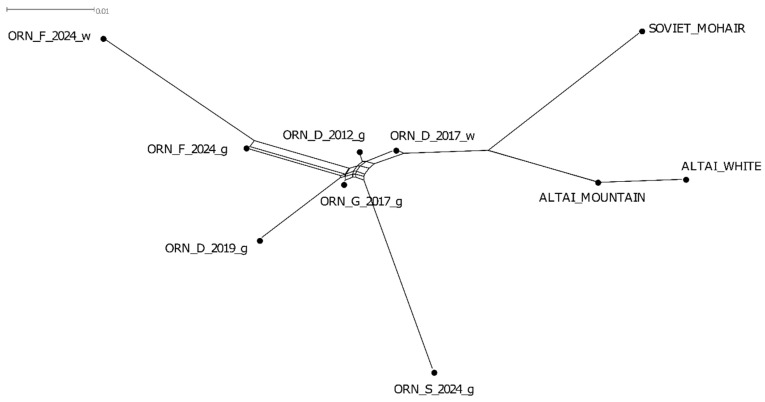
Neighbor-Net graph based on *F*_ST_ distances plotted for the Orenburg populations and three other local goat breeds.

The network analysis based on *F*_ST_ distances showed that the populations of the Orenburg breed were separated from the other goat groups studied (Figure 2). The Altai Mountain, Altai White and Soviet Mohair breeds used as the comparison group were connected to the Orenburg network via the ORN_D_2017_w branch. Considering the Orenburg groups, we found that ORN_F_2024_w, ORN_S_2024_g and ORN_D_2019_g had the longest branches, while the other populations were located near the network edges. In addition, the ORN_F_2024_g and ORN_F_2024_w formed their own cluster within the Orenburg network.

The lowest pairwise *F*_ST_ values were estimated between ORN_D_2012_g and ORN_D_2017_w (*F*_ST_ = 0.004), ORN_D_2012_g and ORN_G_2017_g (*F*_ST_ = 0.004), and ORN_D_2017_w and ORN_G_2017_g (*F*_ST_ = 0.007). The highest *F*_ST_ values were established for the pair of ORN_F_2024_w and ORN_D_2019_g (*F*_ST_ = 0.045) and that of ORN_F_2024_w and ORN_S_2024_g (*F*_ST_ = 0.06).

**Figure 3:**
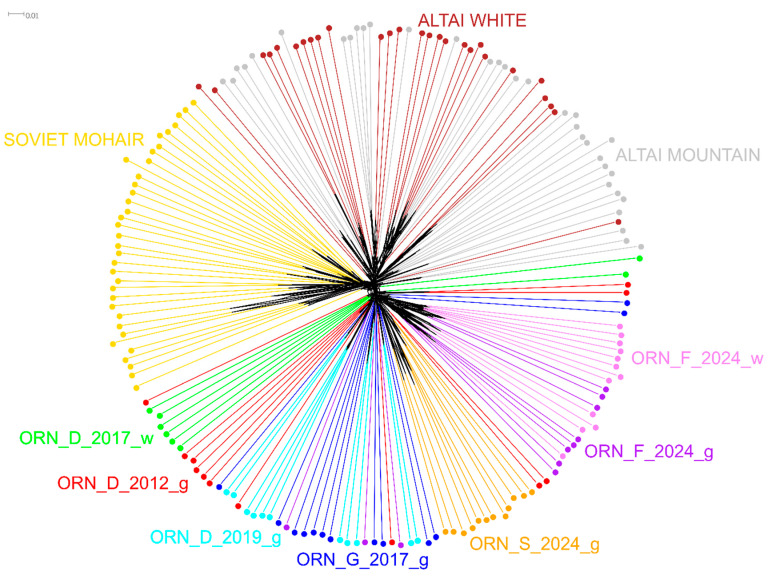
Neighbor-Net graph based on IBS distances built for the Orenburg populations and three other local goat breeds.

The Neighbor-Net analysis based on IBS distances demonstrated that animals from the Orenburg populations predominantly joined the cluster corresponding to their breed (Figure 3). The individuals from ORN_D_2012_g were found in most populations, except for ORN_F_2024_g and ORN_F_2024_w. These two latter groups were differentiated within the Orenburg cluster, except for three individuals from ORN_F_2024_g. In addition, we detected two outliers in ORN_D_2017_w that were located near the Altai Mountain cluster.

### 3.2. PCA and Population Structure Analyses

**Figure 4:**
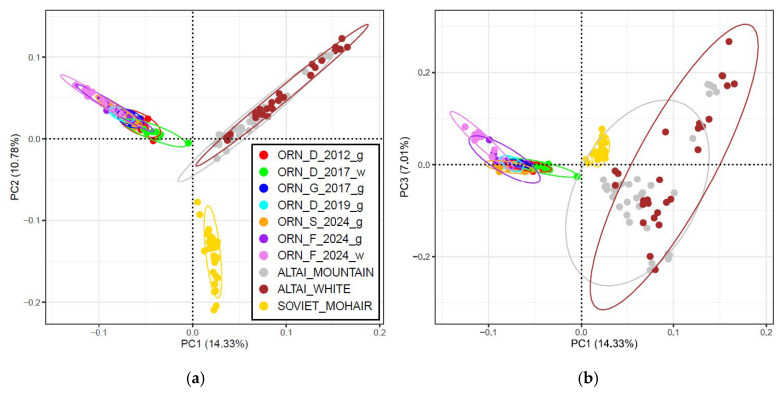
Principal component analysis performed among the Orenburg populations and comparison group breeds studied: (**a**) for the first two principal components (PC1 and PC2); and (**b**) for the first and third principal components (PC1 and PC3).

The PCA performed for all the studied breeds (Figure 4) showed that the first principal component (PC1) separated a cluster including all the Orenburg populations from the comparison group (Figure 4a). Two outliers in ORN_D_2017_w that were found earlier were located near the PC1. The analysis within the PC1–PC3 axes showed the slightest differentiation of ORN_F_2024_w within the Orenburg cluster (Figure 4b).

**Figure 5:**
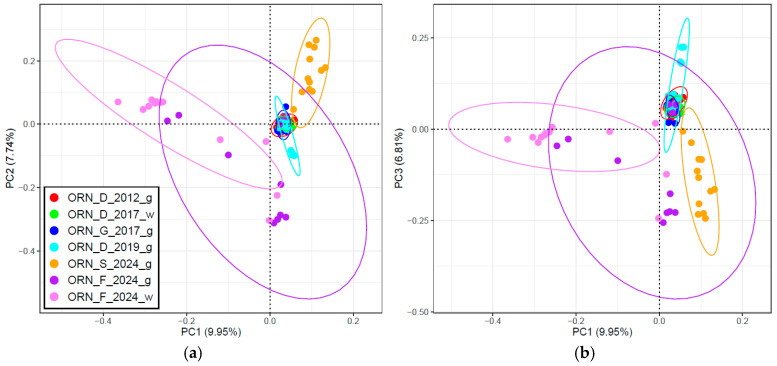
Principal component analysis among populations of the Orenburg breed. The first two principal components (PC1 and PC2) (**a**) and the first and third principal components (PC1 and PC3) (**b**) underwent the analysis.

Based on the PCA performed for populations of the Orenburg breed, PC1 separated the majority of goats from ORN_F_2024_w and three outliers from ORN_F_2024_g from the united large cluster (ULC) (Figure 5). The ULC consisted of ORN_D_2012_g, ORN_D_2017_w, ORN_G_2017_g and ORN_D_2019_g (except for several outliers) and several individuals from ORN_F_2024_g. Amongst all populations, the more complex spatial distribution was observed within ORN_F_2024_g that was divided into three groups. The first one included three outliers separated by PC1, individuals within the ULC, and five goats differentiated from the ULC by PC2 (Figure 5a) and PC3 (Figure 5b). In addition, ORN_S_2024_g was separated from the ULC by PC2 and PC3.

**Figure 6:**
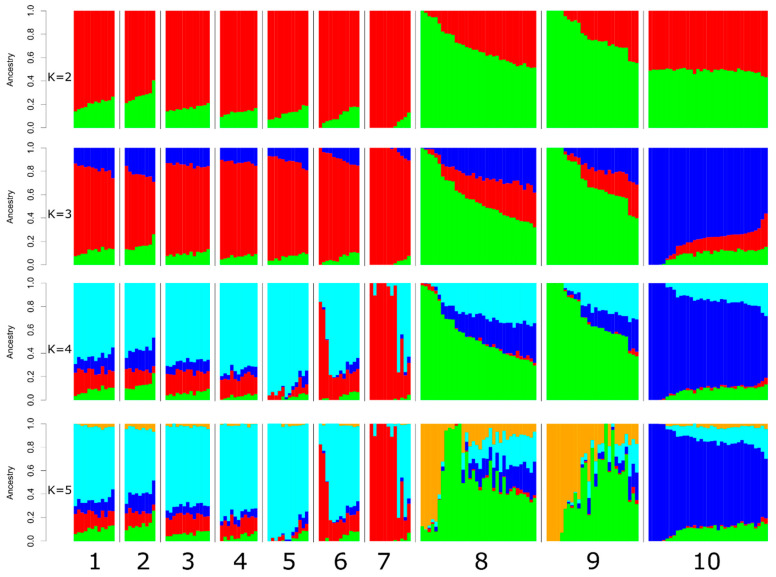
Population structure of the Orenburg goat populations and comparison group breeds. Goat populations: 1, ORN_D_2012_g; 2, ORN_D_2017_w; 3, ORN_G_2017_g; 4, ORN_D_2019_g; 5, ORN_S_2024_g; 6, ORN_F_2024_g; 7, ORN_F_2024_w; 8, ALTAI_MOUNTAIN; 9, ALTAI_WHITE; 10, SOVIET_MOHAIR. Admixture bar plots represent individual ancestry proportions shown in different colors in the studied populations.

Clustering using the Admixture software showed that at K = 2, the population structure of Orenburg goat groups differentiated from those in the Altai Mountain, Altai White and Soviet Mohair breeds (Figure 6). At K = 3, all the Orenburg populations had genetic elements that were shared with the other breeds and were present in different proportions. The lowest CV error was estimated at K = 5. At this K value, we found that the genetic components that were predominant in the Soviet Mohair (blue color) and Altai Mountain (green and orange) breeds were also present in small amounts in the genomes of ORN_D_2012_g, ORN_D_2017_w, ORN_G_2017_g and ORN_D_2019. We observed that ORN_S_2024_g was characterized by a more consolidated population structure compared to the other Orenburg groups. In addition, we detected the presence of an additional ancestral background (red color) that was less obvious in ORN_S_2024_g and predominant in ORN_F_2024_w.

Comparing modern the Orenburg populations and other breeds, we found 48 SNPs, which were above the threshold value (i.e., 0.1% of SNPs with maximum *F*_ST_ values; see Supplementary Table S1). The respective *F*_ST_ values varied from 0.36 to 0.51. The allele frequencies for several SNPs were different in Orenburg goats relative to the comparison group of other breeds (Supplementary Table S1).

### 3.3. Genetic Diversity

An analysis of the genetic diversity indicators showed that *H_O_* varied from 0.401 in ORN_F_2024_w to 0.408 in ORN_D_2012_g and ORN_D_2017_w (Table 2). Estimates of the *uF*_IS_ coefficient that were significant at the 95% confidence level for all populations of the Orenburg breed were positive in ORN_D_2012_g and ORN_D_2017_w and negative in the other groups. Values of the *A_R_* coefficient ranged from 1.945 in ORN_F_2024_w to 1.976 in ORN_D_2017_w.

### 3.4. Pattern of the Runs of Homozygosity Distribution

ROH were found in all the Orenburg goat populations and comparison group breeds (Table 3). The mean ROH length varied from 72.6 Mb in ORN_G_2017_g to 108.9 Mb in ORN_F_2024_w. The lowest mean ROH number was identified in ORN_D_2012_g (28.3) and the largest one was found in ORN_F_2024_w (36.7). Considering specific individual values among Orenburg goats, the maximum ROH length was estimated in ORN_F_2024_w (207 Mb) and the largest ROH number was detected in ORN_D_2017_w (54).

**Figure 7:**
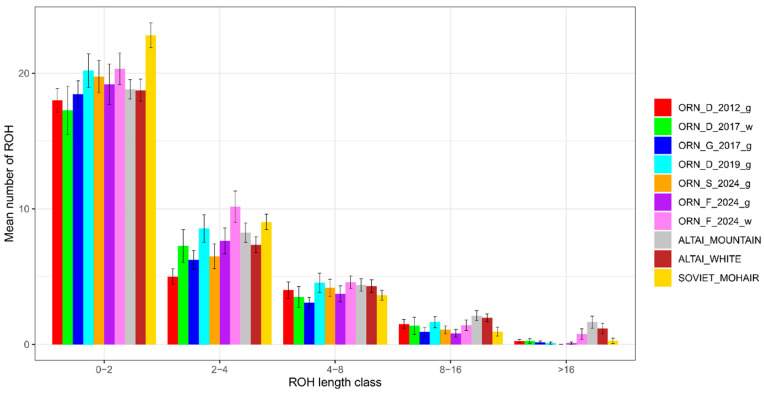
Runs of homozygosity (ROH) distribution in length classes in populations of the Orenburg and other breeds studied.

All the Orenburg populations had a smaller overall number of ROH (467–760) than the other breeds studied (1552–2300). An analysis of the ROH distribution in length classes showed that the shortest ROH (0–2 Mb) were the most frequent in all the studied goat groups and varied from 73.4% to 81.7% in the Orenburg populations and from 73.7% to 78.9% in the comparison group breeds (Figure 7). The longest ROH (>16 Mb) were the rarest and ranged from 0.1% to 0.4% in the Orenburg populations and from 0.4% to 2.8% in the comparison group breeds.

**Figure 8:**
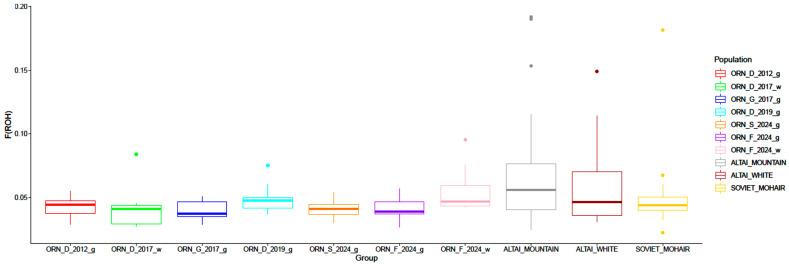
Variation in genomic inbreeding coefficient (*F*_ROH_) based on runs of homozygosity (ROH) in the Orenburg goat populations and other breeds studied.

Differences in *F*_ROH_ values (Figure 8) were lower in the Orenburg populations (*F*_ROH_ = 0.03 to 0.039), except for ORN_F_2024_w (*F*_ROH_ = 0.045). The mean *F*_ROH_ values estimated in the Orenburg populations were compatible with those obtained in the comparison group breeds. However, the individual *F*_ROH_ values were higher in the comparison group breeds (with maximum *F*_ROH_ from 0.14 to 0.18) than in the Orenburg populations (with maximum *F*_ROH_ from 0.04 to 0.09).

## 4. Discussion

Many local goat breeds are in a critical status of conservation, surviving in small herds threatened by the cotemporary demands of intensive commercial production [41]. This vulnerability is greater within breeds that are selected in order to produce non-food products (down or wool is a good example). This is because their maintenance and future prosperity depend on the market demand and are influenced by sociocultural (fashion instability and a decline in the popularity of national traditional clothing), economic (rising costs, a lack of the automation of the production process, and the availability of cheaper imitations), and ecological factors (e.g., climate change). Unfortunately, the Orenburg goat breed is facing a crisis at the present time that might be deepened in future and lead to the extinction of this breed. The last breeding farm, the Donskoy Agricultural Production Company (collective farm), was shut down in 2023 because of decreasing cost efficiency in the rearing of Orenburg goats, and its nucleus flock was sold out to local smallholders. As a result, the quality of breeding work is decreasing significantly, and this will inevitably result in breed degradation.

In this study, we focused on the analysis of the population structure and genetic diversity of modern Orenburg goats in comparison with archived and earlier generations raised in the breeding farms. Uncontrolled gene flow can cause a loss of the genetic purity of breeds [42]. In this regard, we additionally analyzed the probable admixture events that might occur between the Orenburg and other Russian breeds.

Based on PCA and network and admixture analyses, we made two general conclusions. First, the ancestral genetic background that was found in the archived group (ORN_D_2012_g) is still present in all the Orenburg populations, covering sampling periods from 2017 to 2024. To the best of our knowledge, no records and no scientific notes have reported that foreign breeds (Angora, Cashmere, etc.) have improved the Orenburg breed. The previously reported results of the genomic analysis of the population structure of the Orenburg breed in the context of five Mongolian indigenous [43], Asian [44] and other worldwide breeds from the AdaptMap dataset [23] showed no recent admixture events.

Second, we found moderate differentiation within the modern Orenburg populations. These differences cannot be explained by admixture events with the other breeds that were present in the SNP dataset (Altai Mountain, Altai White and Soviet Mohair). In addition, genetic connections between the Orenburg (ORN_D_2017_w, ORN_G_2017_g and ORN_D_2019_g) and Altai Mountain breeds were found in our previous work [23]. This pattern that might have occurred due to their common ancestral background as suggested earlier or due to the presence of admixed animals as found currently (Figure 6) was not revealed in the modern Orenburg populations.

We may hypothesize the possible reasons that underlie this genomic pattern of the differentiation of the modern Orenburg populations. Selection in the Orenburg breed was based on phenotypes that were associated with the quantity and quality of down (color, weight and length of down hair and down yield) [14]. According to the latest review, along with two purebred types (gray and white), the crossbred white goats of F_1_ White Don × White Orenburg are spreading in Orenburg Oblast [14]. The Don breed is characterized by a higher hair yield but by a lower quality and fineness of the down than the Orenburg breed [45]. Several assessments of the down production of Orenburg purebred goats and F_1_ White Don × White Orenburg crosses have been performed [46,47]. Although the down quality is higher in Orenburg purebred goats, the demand for white down for knitting openwork shawls “pautinka” is rising [46]. In this regard, the crossing of purebred gray goats with White Don sires might be performed to obtain a bulk of white goats to meet the need for white down. On the one hand, the Don breed might be crossed with goats from ORN_F_2024_w and ORN_F_2024_g. The values of genetic differentiation might be indirectly interpreted in favor of this theory. Regarding modern populations, *F*_ST_ estimates were 0.06 between ORN_F_2024_w and ORN_S_2024_g, 0.037 between ORN_F_2024_g and ORN_S_2024_g and 0.022 between ORN_F_2024_g and ORN_F_2024_w. On the other hand, during past and the latest expeditions, a sample collection of the Don breed specimens was not successful, so we had no SNP genotypes of the representatives of this breed. Major concerns pertaining to the erosion of genetic resources of the Don breed were raised in 2007 [48], and by 2019, the Don breed was at risk of extinction [16]; worryingly, no records on the status of the breed were available in 2023 [17]. Moreover, ORN_F_2024_w demonstrated the highest levels of genomic inbreeding based on an ROH estimation across the Orenburg populations. Although large-scale crossings with the Don breed are unlikely to have happened, we have no evidence that crossbred individuals have not been found in the flocks in farms, where sampling was performed.

As mentioned in the Section 1, smallholders also breed Orenburg goats in the Republic of Bashkortostan [17]. Due to the decreasing population size of this breed, the rotation of the sires is very likely. We think that it is reasonable to suggest that rotation took place in one farm and did not occur in the other. Moreover, there were once three production types in Orenburg gray goats and two types in white goats [49]. The animals representing different production types may have been raised at different farms.

Along with genetic differentiation, we addressed genetic diversity and ROH distribution. In comparison to our results, *H_O_* varied from 0.288 to 0.292 in Laos-native goats [44], from 0.728 in the Valais to 0.847 in the Styrian Pied [22], and from 0.301 to 0.380 in Canarian breeds [50]. Our findings indicated that all the Orenburg populations displayed compatible or lower *F*_ROH_ values in comparison with those obtained in various local and commercial breeds as summarized below. For example, another fiber-producing breed, the Cashmere raised in Inner Mongolia, had a lower genomic inbreeding coefficient (*F*_ROH_ = 0.026) [51]. Dadousis et al. [18] reported that *F*_ROH_ varied from 0.069 in the Garfagnina to 0.143 in the Girgentana breeds raised in Italy. An analysis of the genomic landscape of inbreeding in the Spanish Florida breed revealed the presence of goats characterized by higher (0.1296) and lower (0.0148) *F*_ROH_ estimates [52]. The genomic inbreeding coefficient was 0.053 in an Andalusian population of the Murciano-Granadina breed [53]. The mean *F*_ROH_ value was 0.061 in Czech and Slovak goats and varied from 0.025 in the White Shorthair to 0.128 in the Valais breed [22]. *F*_ROH_ values ranged from 0.02 to 0.21 in Chinese breeds [54] and from 0.030 to 0.103 in Canarian breeds [50]. In addition, genomic coverage in ROH in the Orenburg populations was higher than in Laos-native goats (5.92–6.85 Mb) [44] and lower than in Chinese breeds (85–257 Mb) [54]. In addition, the identified SNPs (Supplementary Table S1) should be studied further and more precisely because they could be associated with specific phenotypic traits of Orenburg goats and could help in preserving the breed’s genetic diversity.

In recent decades, the initiatives related to livestock breeding have been aimed at increasing agricultural production volumes [55,56]. The usage of several highly productive commercial breeds has led to a decline in population sizes and, consequently, in the loss of the genetic diversity of local breeds [57,58,59]. To preserve these valuable national gene pools, the main objectives of the NCGRAA created in 2024 are the establishment and replenishment of the national bank of especially valuable specimens of livestock genetic resources and ensuring the guaranteed long-term preservation of the functionality of these specimens.

**Figure 9:**
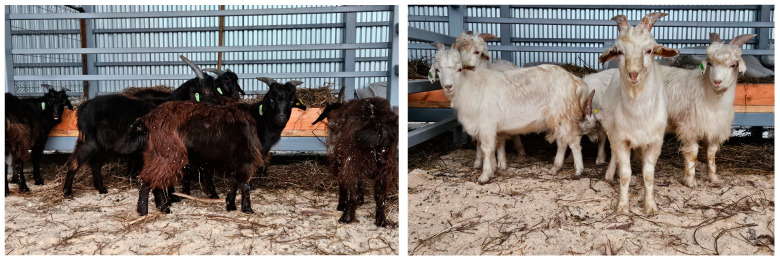
The Orenburg goats brought from Orenburg Oblast to the L. K. Ernst Federal Research Center for Animal Husbandry experimental farm in Moscow Oblast. (Image source: authors’ own photograph).

Since the Orenburg goat breed is facing a crisis, the NCGRAA is taking initial steps in saving this threatened population. Based on the results of this study, the female and male representatives of the Orenburg breed were selected to be donors of embryos and semen for cryopreservation. The goats were brought to the LKEFRCAH experimental farm in Moscow Oblast (Figure 9) to maintain them in situ. In addition, the goats will be bred ex situ.

In future, we plan to perform a whole-genome sequencing of goats of the Orenburg breed from different sampling years in order to enrich the genomic information compared to that already produced in this study.

## 5. Conclusions

Here, we present the results of the most comprehensive study of the gene pool of Orenburg down goats based on SNP genotypes. The modern Orenburg breed populations that represented the sampling in 2024 did not display critical values of genetic diversity. The analysis of ROH distribution patterns showed moderate inbreeding in all the Orenburg goat populations. The study results provided evidence that the ancestral genetic background is still present in all the Orenburg populations, covering sampling from 2017 to 2024. Our findings have been used to select representatives of the Orenburg breed that are included in future conservation programs implemented by the LKEFRCAH NCGRAA to save this breed for future generations.

## Figures and Tables

**Table 1:** Brief description of the populations of the Orenburg goat breed studied.

Population	*n* ^1^	Color	Year of Collection	Farm	Source
ORN_D_2012_g	12	gray	2012	Donskoy (nucleus)	This study
ORN_D_2017_w	8	white	2017	Donskoy (nucleus)	[23]
ORN_G_2017_g	13	gray	2017	Guberlinsky (nucleus)	[23]
ORN_D_2019_g	11	gray	2019	Donskoy (nucleus)	[23]
ORN_S_2024_g	12	gray	2024	Sapinov (smallholder)	This study
ORN_F_2024_g	11	gray	2024	Frolov (smallholder)	This study
ORN_F_2024_w	12	white	2024	Frolov (smallholder)	This study

^1^ *n*, sample size.

**Table 2:** Genetic diversity indicators for the goat populations studied.

Population	*n* ^1^	*H_O_* ^2^	*uH_E_* ^3^	*uF*_IS_ ^4^	*A_R_* ^5^
ORN_D_2012_g	12	0.408	0.409	0.003 [0; 0.006]	1.972
ORN_D_2017_w	8	0.408	0.414	0.012 [0.009; 0.015]	1.976
ORN_G_2017_g	13	0.407	0.406	−0.004 [−0.006; −0.002]	1.968
ORN_D_2019_g	11	0.403	0.396	−0.016 [−0.019; −0.013]	1.958
ORN_S_2024_g	12	0.407	0.389	−0.042 [−0.044; −0.04]	1.947
ORN_F_2024_g	11	0.406	0.397	−0.021 [−0.024; −0.018]	1.962
ORN_F_2024_w	12	0.401	0.381	−0.049 [−0.051; −0.047]	1.945
ALTAI_MOUNTAIN	34	0.401	0.409	0.02 [0.018; 0.022]	1.971
ALTAI_WHITE	27	0.406	0.401	−0.012 [−0.014; −0.01]	1.964
SOVIET_MOHAIR	35	0.404	0.403	−0.001 [−0.003; 0.001]	1.964

^1^ *n*, sample size; ^2^ *H_O_*, observed heterozygosity; ^3^ *_U_H_E_*, unbiased expected heterozygosity; ^4^ *uF*_IS_, inbreeding coefficient based on the difference between *_U_H_E_* and *H_O_* with a 95% confidence interval (CI; in square brackets); ^5^ *A_R_*, allelic richness. Standard errors of the mean were ±0.001 for *Ho*, *uH_E_* and *A_R_*.

**Table 3:** Mean runs of homozygosity (ROH) length and mean ROH number in the Orenburg and other goat populations studied.

Population	*n* ^1^	ROH Length			ROH Number		
		Mean	Min ^2^	Max ^3^	Mean	Min	Max
ORN_D_2012_g	12	79.3 ± 5.3	46.2	99.9	28.3 ± 1.4	22	35
ORN_D_2017_w	8	82.6 ± 15.6	46.7	182.3	29.6 ± 3.8	21	54
ORN_G_2017_g	13	72.6 ± 4.4	40.1	98.1	28.8 ± 1.1	24	35
ORN_D_2019_g	11	93.7 ± 7.9	62.7	159.8	34.2 ± 2.0	26	48
ORN_S_2024_g	12	77.7 ± 4.8	53.2	112.9	30.9 ± 1.8	24	46
ORN_F_2024_g	11	76.9 ± 5.7	41.4	113.4	31.0 ± 1.9	20	41
ORN_F_2024_w	12	108.9 ± 11.6	76.9	207.0	36.7 ± 1.3	29	45
ALTAI_MOUNTAIN	34	137.3 ± 17.8	35.7	442.4	34.8 ± 1.8	18	57
ALTAI_WHITE	27	116.2 ± 13.1	47.9	340.8	33.0 ± 1.5	20	49
SOVIET_MOHAIR	35	91.1 ± 10.0	23.7	409.3	36.0 ± 1.3	14	52

^1^ *n*, sample size; ^2^ Min, minimum; ^3^ Max, maximum.

## Data Availability

The SNP genotypes for the Soviet Mohair, Altai Mountain, Altai White breeds and Orenburg goats (samplings of the years 2017 and 2019) are available at https://figshare.com/articles/dataset/SNP-based_genotyping_provides_insight_into_the_West_Asian_origin_of_Russian_local_goats/14706429 (accessed on 5 September 2024). The SNP genotypes for goats from the archived and modern Orenburg populations used in this study are available on request from the corresponding authors.

## Supplementary Materials

The following supporting information can be downloaded at: https://www.mdpi.com/article/10.3390/genes15111375/s1, Supplementary Table S1: SNPs that differed most significantly between the Orenburg goats (OREN2024) and comparison group.
